# Intermittent pneumatic compression does not improve post-match neuromuscular, biochemical or perceptual recovery in international-level youth soccer players: a randomized placebo-controlled trial

**DOI:** 10.1186/s13102-026-01580-5

**Published:** 2026-02-09

**Authors:** Filipe Maia, Sandro Tito, Marco Correia, Fábio Y. Nakamura, João Ribeiro

**Affiliations:** 1 Research Center in Sports Sciences, Health Sciences and Human Development, University of Maia, Av. Carlos de Oliveira Campos, Maia, 4475-690 Portugal; 2 Polythecinc Institute of Maia Research Center (N2i), Maia, Portugal; 3 Department of Performance Optimization (GOD), Sporting Clube de Braga SAD, Braga, Portugal; 4 School of Sport and Leisure (ESDL-IPVC), Polytechnic Institute of Viana do Castelo, Viana do Castelo, Portugal; 5 Graduate Program in Physical Education, Federal University of Pernambuco, Recife, Brazil; 6 Football Science Institute Lab (FSI Lab), Granada, Spain; 7 SC Braga Education, Sporting Clube de Braga SAD, Braga, Portugal

**Keywords:** Sports, Athletic performance, Compression therapy, Youth athletes

## Abstract

**Background:**

This study aimed to investigate the effectiveness of a single session of high-pressure intermittent pneumatic compression (IPC) on post-match recovery in international level youth soccer players, using neuromuscular, biochemical, and perceptual markers.

**Methods:**

Forty-two observations (across 6 matches) of 23 elite U19 soccer players (age: 17.45 ± 0.72 years; height: 1.82 ± 0.06 m; body mass: 74.95 ± 6.45 kg) participated in this randomized, placebo-controlled trial. Following competitive matches, participants were assigned to receive either 30 min of high-pressure IPC (200 mmHg) or a placebo intervention (hydrant cream). Recovery status was assessed pre-match, and at 30 min, 24- and 48-hours post-match using countermovement jump height, low-frequency fatigue of the knee extensors (Myocene^®^), creatine kinase (CK) concentrations, and self-reported fatigue and soreness. Linear mixed models were used to examine the effects of time, group, and time x group interaction. Covariates such as perceived exertion, GPS metrics, and sleep metrics were included when appropriate.

**Results:**

No significant time x group interactions were found for any of the variables under analysis (*p* = 0.089 to 0.834). Significant main effects of time were detected in CK, perceived fatigue and soreness (*p* < 0.001), confirming match-induced fatigue, but without group differences. Exploratory analyses revealed that internal and external load, as well as total sleep time influenced recovery markers, particularly CK and perceptual responses.

**Conclusion:**

A single session of high-pressure IPC does not seem to enhance post-match recovery in elite youth soccer players compared to a placebo, on neuromuscular, biochemical, and subjective markers.

**Trial registration:**

ClinicalTrials.gov code NCT06636942 (date of registration: 02/10/2024).

## Background

Soccer has evolved substantially in recent decades, with the modern game becoming increasingly faster-paced and physically intense [1]. Modern players engage in a greater frequency of high-intensity actions than ever before, which are often accompanied by less time for recovery due to increasingly dense competitive schedules [2, 3]. In particular, previous evidence demonstrates that match intensity has consistently increased over time, with greater high-intensity running distances (up to 36%), as well as a higher number and distance of high-intensity actions and sprints across all playing positions, indicating a substantial rise in physical demands [1]. Moreover, players frequently engage in competitive events separated by as little as 72 h, with some accumulating up to 80 matches over a 40-week season [3].

Combining these factors leads to heightened physical fatigue, which can impair players’ technical, tactical, and physical performance [4]. Specifically, match-induced fatigue may lead to impaired neuromuscular function and physical performance in the lower limbs (e.g., reduced force production and sprint performance), diminished well-being (e.g., heightened delayed onset muscle soreness, perceived fatigue), and alterations in the biochemical profile (e.g., elevated markers of muscle damage [creatine kinase] and inflammation [interleukin-6, C-reactive protein]), whose kinetics have been demonstrated to persist beyond 72 h post-match [5, 6]. As such, the combination of the high demands of competitive soccer and congested fixtures may require athletes and support staff to adopt strategies to accelerate the recovery of neuromuscular function and, ultimately, mitigate the incidence of non-contact musculoskeletal injuries [7, 8].

While some strategies, such as squad rotation, are effective in managing physical status of players, they are not always feasible [9]. Therefore, enhancing recovery practices becomes critical for sustaining players’ readiness across consecutive matches [7]. Recovery is defined as a time-dependent and multifaceted process, and its interventions aim to return the athlete to their physical and psychological baseline state [10]. It encompasses a wide range of domains, including sleep hygiene (e.g., sleep extension), nutrition and hydration (e.g., carbohydrate and protein intake), various post-exercise practices (e.g., massage therapy, cold water immersion), and psychological recovery techniques (e.g., meditation) [8, 11, 12]. Each domain contributes to the broader recovery process by addressing different physiological or psychological aspects of fatigue. For instance, adequate sleep and nutrition are critical for tissue repair and energy replenishment [12], while psychological techniques can help regulate mood, manage stress, and maintain motivation, thereby contributing to the recovery process [13]. Considering post-exercise recovery techniques, various practices aim to accelerate recovery by supporting physiological processes through additional interventions, including, but not limited to cold exposure (e.g., cold water immersion, cryotherapy) [14, 15] or blood flow enhancement (e.g., compression garments) [16, 17]. Although their isolated effects may be modest, in elite sport—where marginal gains can define performance outcomes—such interventions may be strategically valuable [18].

One increasingly popular tool among athletes, perceived with moderate effectiveness in soccer, is intermittent pneumatic compression (IPC) [19]. IPC devices contain sleeves that cover the limbs (typically the lower limbs), with chambers that apply pressure in a cyclic pattern to promote blood flow by alternately occluding and dilating the blood vessels [16]. Originally developed for medical care, IPC is primarily used to prevent or treat conditions like lymphedema, venous stasis, leg ulcers, and deep vein thrombosis [20, 21]. In athletes, this cyclic compression is believed to accelerate recovery by enhancing blood flow, thereby facilitating the clearance of metabolic waste by-products, potentially reducing muscle soreness, edema and swelling, as well as restoring neuromuscular function faster than passive recovery alone [22]. While most previous studies in athletes have employed moderate pressures [23], high-pressure IPC appears to elicit more pronounced physiological effects [16]. At moderate pressures, compression primarily compresses the blood vessels, improving venous return and facilitating blood flow, by tightening arterial diameter [16]. In contrast, the high pressures not only compress the blood vessels, but may also induce arterial dilation, enhancing arterial perfusion and potentially increasing nitric oxide-mediated vasodilation, thereby amplifying metabolite clearance, reducing edema, and promoting faster neuromuscular recovery [16]. Due to their portability and ease of use, IPC devices allow athletes to incorporate this recovery technique independently, without requiring a therapist, in various settings (e.g., at home, during travel, or at the club facilities) [24].

Despite its growing popularity, research on the effectiveness of IPC as a recovery tool remains scarce, especially concerning its application within highly competitive athletes. Such athletes exhibit unique physiological characteristics that set them apart from recreationally active individuals or lower-level athletes, including anthropometric, biochemical, physiological, genetic, among many others [25]. Therefore, caution should be exercised when extrapolating results from studies conducted with lower-level athletes, as their recovery needs and physiological responses may differ significantly from those of elite athletes [26].

Aiming to address this gap in the scientific literature, our study investigates the effectiveness of a single session of high-pressure IPC on post-match recovery in high-level youth (under-19) soccer players, under ecological scenarios (i.e., following a soccer match). This study integrates objective and subjective indicators of recovery, including neuromuscular performance (low-frequency fatigue [LFF] and countermovement jump [CMJ]), perceptual (perceived soreness and perceived fatigue], and biochemical markers of muscle damage (creatine kinase concentrations).

## Methods

### Participants

Twenty-three male Under-19 soccer players (Tier 4 according to the definition of McKay et al. [26]) were included in this study (age: 17.45 ± 0.72 years; height: 1.82 ± 0.06 m; body mass: 74.95 ± 6.45 kg). A total of 42 observations (21 IPC and 21 placebo) were collected in a counterbalanced and randomized manner, as some players were assessed more than once during the competitive season. To be eligible to participate in this study, participants were required to play at the highest national level (national first division and European Youth League) and compete internationally, be male, free from musculoskeletal injuries during the current season, and have played more than 70 min on the official match, as used elsewhere [27]. Goalkeepers were excluded from this study due to their different match demands. Importantly, all players participated a maximum of 2 occasions in the study. This study was approved by the University of Maia Ethics Committee (project 97/2022), and written informed consent was obtained from all athletes prior to participation, and, when applicable, from their legal guardians (< 18 years old). The study protocol was registered on ClinicalTrials.gov (NCT06636942).

Due to the club’s policy of granting players a day off following a match and participants’ personal commitments, this study has some missing data that must be acknowledged. Specifically, 11 observations are missing at the post-match time point (~ 25%), 22 observations at the 24-hour time-point (~ 50%); and 12 observations at the 48-hour assessment (~ 25%).

### Experimental design

This study took place during two in-season periods of the Portuguese competitive calendar. Participants underwent four assessment time points: 2 hours pre-match (MD −2 h), 30 minutes following the match (+ 30’ MD), and at 24 h (MD + 1) and 48 h (MD + 2) post-match.

**Fig. 1:**
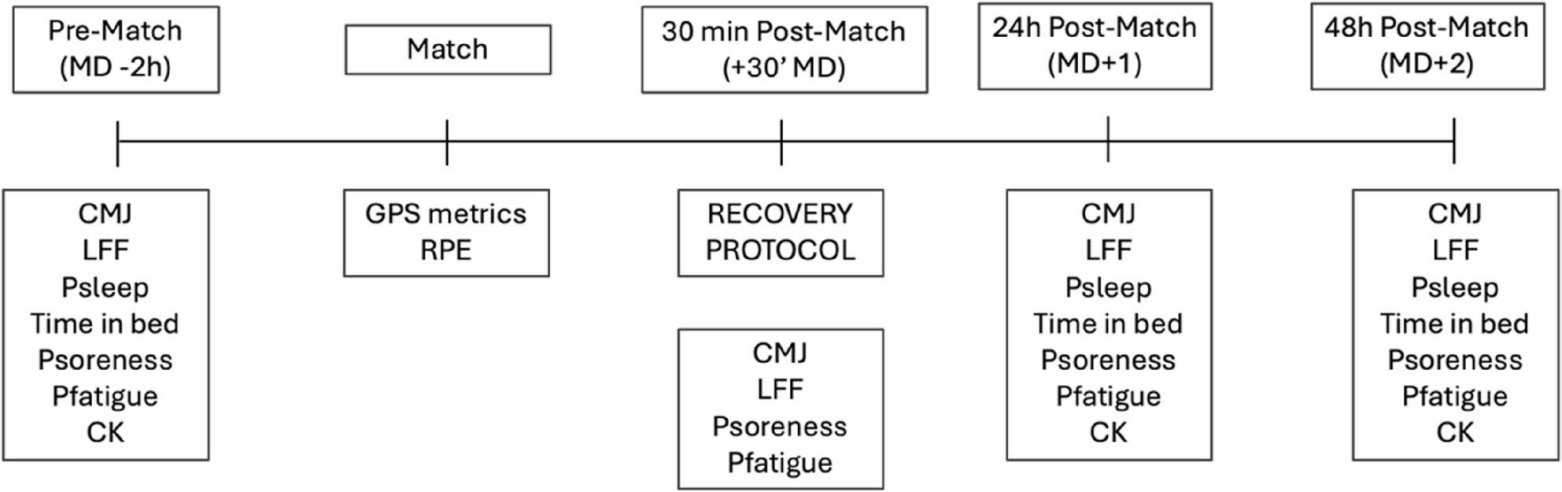
Study procedures and timeline. Notes: CMJ – countermovement jump; LFF – low-frequency fatigue; Psleep – perceived sleep quality; Psoreness – perceived soreness; Pfatigue – perceived fatigue; Time in bed – total time in bed aimed at sleep; RPE – rating of perceived exertion; Recovery protocol – either intermittent pneumatic compression or placebo; CK – creatine kinase

Following the soccer match, the participants who completed at least 70 min of the match were randomly assigned to either IPC or placebo group. Muscular function was assessed with countermovement jump height (CMJ), and LFF. Subjective questionnaires were also employed: perceived fatigue (PFatigue), perceived soreness (Psoreness), perceived sleep quality (Psleep) [28], and time in bed. These measures were recorded individually upon the athletes’ arrival at the data collection site, with players completing the questionnaires independently and without access to other players’ responses. Finally, biochemical measurements of creatine kinase (CK) were also conducted. The schematic representation of study design and timeline is described in Fig. 1. The study was conducted in accordance with the Consolidated Standards of Reporting Trials (CONSORT) guidelines [29].

A standardized warm-up was carried out prior to the CMJ assessments at MD-30’, MD + 1, and MD + 2, consisting of 10 squats and 5 submaximal CMJs, with 2 min of passive recovery between each exercise.

To minimize bias from external influences, participants were instructed to refrain from physical activity outside the club and maintain their nutritional and sleep habits during the data collection period. Most daily meals were provided and consumed at the club’s facilities under the supervision of the club’s nutritionist, ensuring a consistent dietary intake throughout the protocol. Adherence to regular routines was monitored by the team’s sport scientist.

#### Countermovement jump

The CMJ height was recorded across three attempts, with a one-minute rest interval between each. Measurements were conducted using a Chronojump mat (A3 size, Boscosystem, Barcelona, Spain) operating at a frequency of 1000 Hz. The best (highest) jump was used for the analysis [30]. During each attempt, participants were instructed to keep their hands on their hips and jump as high as possible, selecting their own preferred countermovement depth. All athletes were familiar with this procedure, as it is routinely used in their training sessions, and the same setup and instructions were applied consistently across all testing sessions to minimize potential variations (e.g., jump strategy).

#### Low-frequency fatigue

Low-frequency fatigue was assessed using Myocene (Myocene, Liège, Belgium). Participants were seated with their hip and knee joints positioned at a 90º angle, and their leg in contact with a custom-built dynamometer (Myo-sensor). The Myo-sensor recorded the evoked forces at a rate of 4 kHz. Muscle electrical stimulation was applied using pre-programmed trains of the Myocene software, with 3 electrodes placed on the quadriceps: a 5 × 10 cm cathode placed transversely over the proximal portion of the rectus femoris, and two 5 × 5 cm anodes positioned over the distal areas of the vastus medialis and vastus lateralis. Sixteen sets of electrical stimuli were delivered, with a 5-second rest between sets. Each set consisted of a single pulse, followed by a train of 5 low-frequency stimuli (20 Hz) and then a train of 18 high-frequency stimuli (120 Hz). The intensity of stimulation began at 25 mA and was incrementally increased by 1 mA after each set, reaching 40 mA by the final set. The Myocene software calculated the ratio of low- to high-frequency evoked forces for each set, with the median of the 16 ratios (one per set) representing the final output (Powerdex). This evaluation was randomly conducted on both legs, and each protocol had a total duration of 2 min [27, 31].

#### Subjective measurements

Perceptual responses were assessed using the CR-10 rating of perceived exertion scale (RPE) following the match, as a measurement of internal load. Questionnaires of perceived sleep quality, perceived fatigue and perceived soreness were also used (scale range: 1 to 7) [28], as well as self-reported time in bed (aiming to sleep).

#### Creatine kinase measurement

Serum creatine kinase (CK) concentrations were determined using a spectrophotometer (Vario II Photometer, Diaglobal, Germany) with a commercial reagent kit (CK321, Diaglobal, Germany). Capillary blood samples were obtained from the fingertip using a sterile single-use lancet. For CK analysis, 20 µL of capillary blood were collected into heparinized capillary tubes. CK activity was evaluated enzymatically by quantifying the rate of NADPH production, based on absorbance readings at 340 nm, and expressed in units per liter (U/L) at 37 °C.

Hematocrit was assessed using the same spectrophotometric device with a dedicated reagent kit (HCT142, Diaglobal, Germany), based on a separate 10 µL capillary blood sample. Baseline Hematocrit values were used to correct CK concentrations to account for inter-individual differences in plasma volume. All analyses were performed in accordance with the manufacturer’s guidelines.

Importantly, the CK assessment was not conducted immediately following the recovery intervention, due to the delayed kinetic response of CK to exercise-induced muscle damage [32].

#### Match running demands

In addition to the individual match exposure (in minutes), external load variables were recorded using Global Positioning Systems (GPS) with a sampling frequency of 10 Hz (JOHAN Sports, Netherlands). These variables included total distance, high-speed running (HSR) defined as speeds of ≥ 19.8 km/h, sprint distance at ≥ 25.2 km/h, as well as the number of sprints, accelerations (defined as > 3 m/s²), and decelerations (defined as >−3 m/s²). Velocity thresholds were established following the criteria set by Rampinini et al. [33]. Each player wore a GPS unit secured in a manufacturer-specific vest, positioned on the upper back, between the scapulae. On average, horizontal positional accuracy was 390 ± 38.7 mm, with 11.9 ± 3.1 satellites acquired during the recorded matches.

### Intermittent pneumatic compression

Thirty minutes following the soccer match, participants carried out the recovery intervention. IPC was applied to the entire lower limbs, using AirRelax Leg Recovery System Plus (AirRelax, California, USA), with participants lying on a stretcher. The device consisted of four air chambers that applied cyclic pressure to the limbs. A 30-minute peristaltic protocol was used, in which the four air chambers inflated sequentially. The inflation sequence alternated between proximal-to-distal and distal-to-proximal directions across cycles, with each chamber inflating individually, maintaining pressure, and then deflating simultaneously at 200 mmHg. Two sleeve sizes were available, and the appropriate size was individually selected for each participant to ensure optimal fit and effective pressure application.

### Placebo intervention

The placebo intervention involved the application of an over-the-counter hydrating cream (unlabeled bottle), free from active substances known to influence recovery, on participants’ quadriceps, hamstrings and calf muscles. Upon placing the cream, participants lay on a stretcher for 30 min. As a credibility-enhancing strategy, participants were informed that they were testing a novel recovery cream that aimed to provide relaxation and reduce muscle soreness. This process was conducted by the principal investigator.

### Statistical analysis

Statistical analyses were conducted using R in RStudio (version 2025.05.0 + 496). A principal component analysis was initially performed on the external load variables obtained from GPS to reduce dimensionality and avoid multicollinearity. Only the first principal component was retained, as it accounted for 48.6% of the total variance, and it was included as a covariate in relevant models.

Linear mixed-effects models were used to analyze all dependent variables. For each outcome, multiple candidate models were tested with different combinations of fixed effects and covariates, and the final model was selected based on the lowest Akaike Information Criterion, supported by additional model fit indices and visual inspection of residual plots. All final models included group, time point, and their interaction as fixed effects, and a random intercept for participant ID. Covariates such as perceived sleep quality, time in bed, perceived exertion, and the principal component of external load were included when appropriate. All models were estimated using Restricted Maximum Likelihood (REML). Type III ANOVA was applied to test the significance of fixed effects.

Post-hoc pairwise comparisons were performed using the Bonferroni correction to evaluate changes across all parameters over time, both within and between conditions. To compare physical demands across matches, a non-parametric Kruskal-Wallis test was performed. Effect sizes are reported as partial eta-squared (η²p) for linear mixed models and as epsilon-squared (ε²) for the Kruskal-Wallis test, with the following interpretations: ε²/η²*p* ≥ 0.01 – small effect, ε²/η²*p* ≥ 0.06 – moderate effect, and ε²/η²*p* ≥ 0.14 – large effect [34]. For pairwise comparisons, effect sizes are reported as Cohen’s d, interpreted as follows: d < 0.20 – trivial effect, 0.20 ≤ d < 0.50 – small effect, 0.50 ≤ d < 0.80 – moderate effect, and d ≥ 0.80 – large effect [35]. The significance level was established at *p* < 0.05.

Test-retest reliability of all physical and physiological measures was assessed across baseline sessions using intra-individual coefficients of variation (CV) and intraclass correlation coefficients (ICC). Coefficients of variation were interpreted as low (< 5%), moderate (5–10%), or high (> 10%) variability, while ICC values were interpreted following commonly accepted thresholds (ICC ≥ 0.75: excellent, 0.60–0.74: good, 0.40–0.59: fair, < 0.40: poor) [36]. Perceptual outcomes were excluded from these analyses due to their ordinal nature. A sensitivity analysis was performed to determine the smallest effect size detectable with the available sample size.

## Results

In total, six matches were analyzed in this study, which did not significantly differ on key metrics (*p* = 0.15 to 0.789) (Table 1).

**Table 1:** Synthesis of match demands

	Match 1 (*n* = 9)	Match 2 (*n* = 7)	Match 3 (*n* = 8)	Match 4 (*n* = 5)	Match 5 (*n* = 8)	Match 6 (*n* = 5)	ε² = 0.14 (*p*-value)
Match exposure (min)	84.7 ± 16.0	90.0 ± 12.1	93.8 ± 9.05	91.3 ± 12.5	92.2 ± 12.0	87.4 ± 4.9	ε² = 0.14(*p* = 0.229)
Total distance (m)	9198 ± 1591	9334 ± 2186	9647 ± 1285	7741 ± 2899	9624 ± 1067	8693 ± 430	ε² = 0.10 (*p* = 0.457)
HSR distance (m)	1644 ± 440	1543 ± 566	1606 ± 412	1402 ± 553	1680 ± 275	1540 ± 191	ε² = 0.05 (*p* = 0.789)
Sprint distance (m)	101.0 ± 46.4	58.2 ± 47.0	115.0 ± 103.0	113.0 ± 88.6	162.0 ± 96.4	94.3 ± 16.1	ε² = 0.12 (*p* = 0.352)
Acc (> 3 m.s) (n)	33.7 ± 9.54	32.2 ± 10.7	41.7 ± 16.1	26.9 ± 10.5	41.8 ± 17.7	32 ± 1.43	ε² = 0.14 (*p* = 0.245)
Decc (>−3 m.s) (n)	48.0 ± 9.8	35.4 ± 10.7	48.4 ± 14.7	35.5 ± 15.3	43.8 ± 10.9	40.4 ± 2.22	ε² = 0.17 (*p* = 0.150)

*Acc *acceleration, *Decc *deceleration, *HSR *high speed running,* ε² *epsilon squared

*P*-value refers to the non-parametric (Kruskal-Wallis) Anova comparing the demands of the different matches

Regarding CMJ height, no significant differences were observed for time (η²*p* = 0.04; *p* = 0.679), group (η²*p* = 0.01; *p* = 0.180), nor time × group (η²*p* = 0.02; *p* = 0.661). A significant effect was observed for the principal component variable (derived from GPS metrics), suggesting that this latent factor significantly contributes to the variance in CMJ height (η²*p* = 0.15; *p* = 0.006).

In respect to LFF scores for non-dominant limb, no significant differences were observed for time (η²*p* = 0.04; *p* = 0.699), group (η²*p* = 0.03; *p* = 0.480), or time × group (η²*p* = 0.08; *p* = 0.0893). Likewise, for the dominant limb, no significant differences were detected for time (η²*p* = 0.001; *p* = 0.889), group (η²*p* = 0.0001; *p* = 0.752), or time × group (η²*p* = 0.01; *p* = 0.834).

Considering CK concentrations, a significant effect of time was observed (η²*p* = 0.59; *p* < 0.001); however, no significant effects were found for group (η²*p* = 0.01; *p* = 0.562) or time × group (η²*p* = 0.05; *p* = 0.237). Specifically, significant differences were observed between baseline values and MD + 1 (d = 1.69; *p* < 0.001), as well as between MD + 1 and MD + 2 (d = 1.02; *p* < 0.001).

For perceived soreness, a significant effect of time was detected (η²*p* = 0.74; *p* < 0.001), but no significant effects of group (η²*p* = 0.02; *p* = 0.567) or time × group (η²*p* = 0.04; *p* = 0.262) were identified. Specifically, significant differences were observed between pre-match and post-match (d = 3.12; *p* < 0.001), pre-match and MD + 1 (d = 1.32; *p* < 0.001), post-match and MD + 1 (d = 1.47; *p* < 0.001), post-match and MD + 2 (d = 2.41; *p* < 0.001) as well as between MD + 1 and MD + 2 (d = 0.77; *p* = 0.011). While non-significant, a moderate effect was observed between pre-match and MD + 2 (d = 0.62; *p* = 0.069). Additionally, RPE showed a significant effect (η²*p* = 0.12; *p* = 0.026), suggesting that perceived match exertion may contribute to the kinetics of perceived soreness.

A significant effect of time was observed (η²*p* = 0.80; *p* < 0.001) for perceived fatigue, but no significant effect of group (η²*p* = 0.007; *p* = 0.766) or time × group were detected (η²*p* = 0.05; *p* = 0.159). In particular, significant differences were observed between pre-match and post-match (d = 4.34; *p* < 0.001), pre-match and MD + 1 (d = 1.36; *p* < 0.001), pre-match and MD + 2 (d = 0.75; *p* = 0.042), post-match and MD + 1 (d = 2.14; *p* < 0.001), post-match and MD + 2 (d = 3.31; *p* < 0.001), as well as between MD + 1 and MD + 2 (d = 0.72; *p* = 0.010). Rating of perceived exertion showed a significant effect (η²*p* = 0.22; *p* = 0.003), suggesting that perceived exertion may contribute to changes in perceived fatigue.

For Tbed, a significant effect of time was detected (η²*p* = 0.16; *p* = 0.003), but no significant effects were found for group (η²*p* = 0.07; *p* = 0.107) or time × group (η²*p* = 0.02; *p* = 0.553). In particular, athletes appear to have slept longer at baseline than at 48 h post-match (d = 0.77; *p* = 0.007). For sleep quality, no significant effects of time (η2p = 0.02; *p* = 0.554), group (η²*p* = 0.03; *p* = 0.101), or time × group (η²*p* = 0.03; *p* = 0.398) were identified.

Data reliability was examined for all objective measures across baseline sessions. Specifically, CMJ demonstrated a CV of 3.6% and an ICC of 0.85 (*p* < 0.001). Low-frequency fatigue, assessed via Myocene, showed CVs of 4.1% and 3.4%, with ICCs of 0.62 (*p* = 0.005) and 0.75 (*p* = 0.005) for the dominant and non-dominant limbs, respectively. Creatine kinase (CK) exhibited a CV of 10.7% and an ICC of 0.72 (*p* < 0.001). Finally, a sensitivity analysis was performed for the variable muscle soreness to determine the smallest effect size that could be detected with the available sample size. This analysis indicated that the study was sufficiently powered to detect moderate-to-large effects between groups (d ≥ 0.70). Observed differences between conditions, however, were mostly trivial, indicating negligible effects of the interventions on recovery outcomes.

**Fig. 2:**
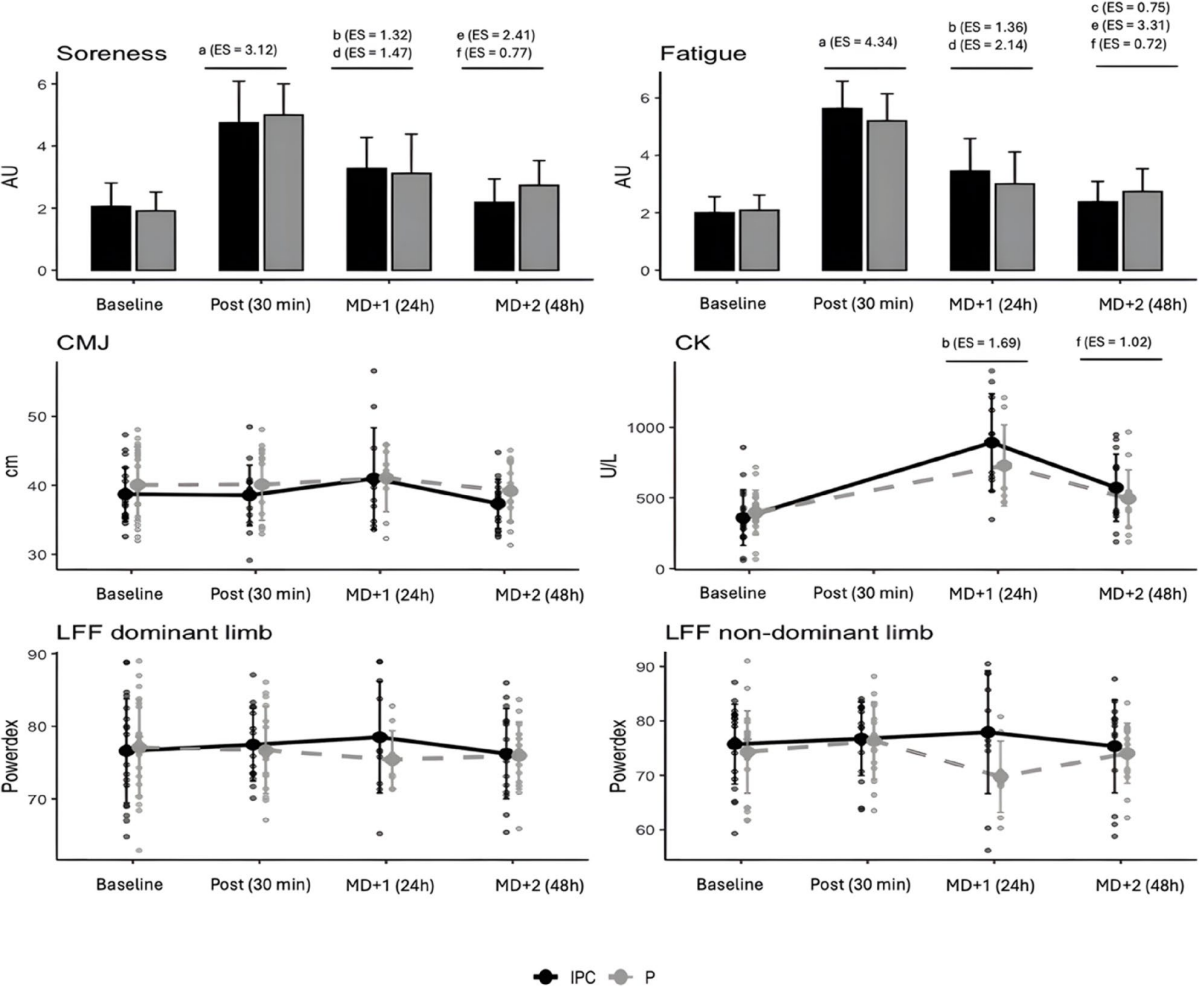
Graphical summary of the results. Notes: IPC – Intermittent Pneumatic Compression, P – Placebo, CMJ – countermovement jump (height), LFF – low-frequency fatigue, CK – creatine kinase, U/L – units per liter, AU – arbitrary units, cm – centimeter, ES – effect size. In particular, it represents: a – significant time differences between pre-match and post-match, b – significant time differences between pre-match and MD + 1, c – significant time differences between pre-match and MD + 2, d – significant time differences between post-match and MD + 1, e - significant time differences between post-match and MD + 2, f – significant time differences between MD + 1 and MD + 2. The bigger dot represents the mean and bars are standard deviation. Individual observations are represented as small dots

A graphical summary of the main results is displayed in Fig. 2.

## Discussion

The aim of this study was to assess the effects of a 30-minute, high-pressure IPC protocol on the recovery kinetics of neuromuscular, biochemical and perceptual responses of international-level youth soccer players. The main findings of this study suggest that IPC, while commonly valued and used by soccer players [19], does not confer superior recovery benefits compared to a placebo, following competitive soccer matches. Despite the expected time-related changes observed in creatine kinase levels, perceived fatigue, and muscle soreness, no significant group or time × group effects were detected for any outcome measure. Importantly, the matches induced measurable exercise-induced muscle damage and fatigue in the players, as evidenced by the time-related increases in creatine kinase levels, perceived muscle soreness, and perceived fatigue, alongside decreases in perceived recovery. These changes confirm that the exercise stimulus was sufficient to elicit post-match fatigue, thereby providing a meaningful context for assessing the efficacy of the IPC intervention.

Intermittent pneumatic compression is thought to accelerate recovery primarily by enhancing blood flow through cyclic compression of the limbs, potentially facilitating the removal of metabolic by-products, reducing muscle swelling, and promoting faster restoration of neuromuscular function [16, 22]. While the scientific literature remains inconsistent on the use of IPC, and sparse concerning elite athletes and intermittent sports, our results are compatible with previous evidence on the topic. Specifically, they align with the findings of Collins et al. [37], who assessed the recovery kinetics of team sport athletes following high-intensity exercise and also observed the absence of significant differences in CMJ height, CK activity, and perceived muscle soreness. Moreover, studies conducted in different populations and exercise modalities have reported similarly limited effects of IPC on neuromuscular recovery. Examples include Wiecha et al. [38] following eccentric exercise, Overmayer and Driller [39] after cycling sessions, Maia et al. [40] in response to flywheel resistance training, and Hoffman et al. [41], following an ultramarathon race. Collectively, these studies reinforce the notion that IPC may have limited efficacy in accelerating neuromuscular recovery, regardless of the type of exercise performed.

The absence of differences in CMJ height observed in our study may reflect not only the ineffectiveness of IPC in accelerating recovery, but also the limited sensitivity of CMJ height to detect subtle changes in neuromuscular fatigue [42]. While CMJ height is commonly used as a metric of fatigue, especially in team sports, it primarily reflects explosive power and may not capture other relevant components of fatigue, such as peripheral impairments or central drive reduction. Moreover, the absence of force platform data limits the ability to assess alternative neuromechanical metrics, which represents a methodological limitation.

Low-frequency fatigue is emerging as a promising tool to measure peripheral fatigue in sports, and previous evidence has highlighted its sensitivity to detect fatigue among soccer players [27]. However, no impairments were observed in our study. One plausible explanation is that the magnitude of peripheral fatigue accumulated during the match, although substantial, may not have reached the threshold necessary to elicit quantifiable changes in LFF. Factors such as tactical formation, or role-specific workload distribution might have influenced the physiological stimulus differently compared to prior findings [27]. Additionally, the mechanisms through which IPC exerts its effects (i.e., blood flow enhancement) may not directly influence the peripheral impairments associated with LFF, which could further explain the lack of differences between conditions. Future studies may benefit from more frequent time-point measurements and consideration of peripheral versus central fatigue dimensions.

Previous research has highlighted that IPC seems to be effective in reducing muscle soreness [23, 38]. This potential benefit may stem from several physiological mechanisms. The intermittent compression may enhance regional blood flow, thus aiding in the clearance of exercise-induced metabolic waste [16, 22]. Concurrently, the external pressure may reduce local inflammation and interstitial fluid buildup, contributing to decreased tissue swelling [22]. Another plausible mechanism involves the stimulation of cutaneous and deep tissue mechanoreceptors, which can modulate or alter pain perception through central and peripheral pathways. These effects resemble those observed with manual therapies like massage, where mechanical stimuli promote relaxation and reduce muscle tone, possibly by decreasing spinal motor neuron excitability [43]. However, perceptual responses are notoriously sensitive to expectancy and contextual cues, raising the possibility that our placebo intervention—which was designed to be credible—reduced the contrast between groups. Additionally, elite athletes may differ from recreational populations in terms of cardiovascular efficiency, tissue resilience, and pain threshold [25], all of which could modulate the perceived impact of a recovery modality. It is also possible that the effects of IPC vary across individuals, with certain athletes responding more favorably than others based on physiological, psychological, or genetic traits. This interindividual variability, commonly referred to as “responders vs. non-responders,” could have diluted any group-level effects in the current sample. Identifying such response patterns in future studies may help tailor recovery strategies more effectively.

While the measurement of perceived fatigue is less prevalent among the scientific literature of IPC, we opted to measure it to include a more holistic view of the recovery kinetics of soccer players. In particular, we hypothesize that similar mechanisms as those observed with perceived soreness are influencing perceived fatigue responses.

Muscle damage was assessed using the biomarker creatine kinase (CK), which is known to peak between 24 and 48 h post-exercise [32], consistent with our findings. This elevation in CK is consistent with previous reference values highlighted in literature, and likely related to the physical demands of the match, including frequent accelerations, decelerations, and sprints [44]. Although we hypothesized that IPC would attenuate the rise in CK levels by enhancing blood flow and facilitating the clearance of metabolic by-products, this effect was not observed. Possible explanations may include the relatively short duration of the IPC protocol (30 min), suboptimal pressure settings, fixed pressure level, or limited physiological impact of a single recovery session. Notably, the literature on the effects of IPC on CK clearance remains limited, mixed, and inconclusive [23], reinforcing the need for future research examining dose-response effects and repeated application strategies over time.

### Limitations

Despite the relevance of the findings presented in this study, several limitations should be acknowledged. Firstly, although the total number of repeated-measures observations was 42, these corresponded to repeated measurements from only 23 participants. However, this was addressed by including participant ID as a random effect in the mixed-effects models, which accounts for the within-subject dependency of the data. This approach should allow for valid inference by modelling the hierarchical structure of the dataset and controlling for individual variability [45]. As such, the increased number of observations should not compromise the independence assumption, and the analytical approach mitigates the risk of inflated Type I errors due to repeated measures. Secondly, the study was conducted following official soccer matches, which, although enhancing ecological validity, limits our ability to standardize the physical load imposed on each player. Moreover, our sample consisted exclusively of international-level male youth soccer players, limiting generalizability to other levels, age groups, or genders.

In addition, the follow-up period was limited to 48 h post-match due to practical and organizational constraints associated with working in a high-performance environment, including restricted access to players and authorization from the technical staff. While the 24–48 h window represents a critical period in which recovery strategies are most commonly applied, extending assessments to 72 h post-match, consistent with international competition regulations and typical match scheduling, could provide further insight into recovery kinetics and the time course of performance restoration. Also, these constraints further precluded the inclusion of more invasive neuromuscular assessments, such as direct measures of muscle force, which should be considered in future research.

Finally, the relatively small sample size may limit the statistical power and requires cautious interpretation of the results. Nonetheless, the inclusion of internationally competing athletes in applied field settings enhances the relevance of these findings for performance practitioners.

## Conclusion

The main findings of this study do not support the use of a 30-minute high-pressure (200 mmHg) IPC protocol as a superior post-match recovery strategy in international-level youth soccer players. No differences were observed compared to a placebo across neuromuscular, biochemical, and subjective markers. However, substantial time-related changes for creatine kinase, perceived fatigue, and soreness confirm the substantial physiological load induced by the competitive matches assessed. Interestingly, perceived exertion, total time in bed, and GPS metrics emerged as relevant modulators of recovery kinetics, particularly influencing muscle soreness, fatigue, and CK responses. These findings highlight the multifactorial and individualized nature of post-match recovery in highly competitive football and suggest that context-specific monitoring of load and recovery may be equally or more impactful than isolated recovery modalities. Future studies should explore these interactions in larger cohorts and across different competitive contexts.

## Data Availability

The datasets originated from the current research are available from the corresponding author on reasonable request.

## Abbreviations

CK: Creatine–kinase
CMJ: Countermovement jump
GPS: Global positioning system
Hz: Hertz
IPC: Intermittent pneumatic compression
Kg: Kilogram
LFF: Low–frequency fatigue
m: meter
MD: Match Day
mmHg: milimeters of mercury
Pfatigue: Perceived fatigue
Psleep: Perceived sleep
Psoreness: Perceived soreness
Tbed: Time in bed
